# Three Capsular Polysaccharide Synthesis-Related Glucosyltransferases, GT-1, GT-2 and WcaJ, Are Associated With Virulence and Phage Sensitivity of *Klebsiella pneumoniae*

**DOI:** 10.3389/fmicb.2019.01189

**Published:** 2019-05-28

**Authors:** Ruopeng Cai, Gang Wang, Shuai Le, Mei Wu, Mengjun Cheng, Zhimin Guo, Yalu Ji, Hengyu Xi, Caijun Zhao, Xinwu Wang, Yibing Xue, Zijing Wang, Hao Zhang, Yunhe Fu, Changjiang Sun, Xin Feng, Liancheng Lei, Yongjun Yang, Sadeeq ur Rahman, Xiaoyun Liu, Wenyu Han, Jingmin Gu

**Affiliations:** ^1^Key Laboratory of Zoonosis Research, Ministry of Education, College of Veterinary Medicine, Jilin University, Changchun, China; ^2^Department of Microbiology, Army Medical University, Chongqing, China; ^3^Institute of Analytical Chemistry and Synthetic and Functional Biomolecules Center, College of Chemistry and Molecular Engineering, Peking University, Beijing, China; ^4^Department of Clinical Laboratory, The First Hospital of Jilin University, Changchun, China; ^5^College of Veterinary Sciences and Animal Husbandry, Abdul Wali Khan University, Mardan, Pakistan; ^6^Department of Microbiology, School of Basic Medical Sciences, Peking University Health Science Center, Beijing, China; ^7^Jiangsu Co-Innovation Center for the Prevention and Control of Important Animal Infectious Disease and Zoonose, Yangzhou University, Yangzhou, China

**Keywords:** *Klebsiella pneumoniae*, phage resistance, virulence, glucosyltransferase (GT), WcaJ

## Abstract

*Klebsiella pneumoniae* (*K. pneumoniae*) spp. are important nosocomial and community-acquired opportunistic pathogens, which cause various infections. We observed that *K. pneumoniae* strain K7 abruptly mutates to rough-type phage-resistant phenotype upon treatment with phage GH-K3. In the present study, the rough-type phage-resistant mutant named K7R^R^ showed much lower virulence than K7. Liquid chromatography-tandem mass spectrometry (LC-MS-MS) analysis indicated that WcaJ and two undefined glycosyltransferases (GTs)- named GT-1, GT-2- were found to be down-regulated drastically in K7R^R^ as compared to K7 strain. *GT-1*, *GT-2*, and *wcaJ* are all located in the gene cluster of capsular polysaccharide (CPS). Upon deletion, even of single component, of *GT-1*, *GT-2*, and *wcaJ* resulted clearly in significant decline of CPS synthesis with concomitant development of GH-K3 resistance and decline of virulence of *K. pneumoniae*, indicating that all these three GTs are more likely involved in maintenance of phage sensitivity and bacterial virulence. Additionally, K7R^R^ and GT-deficient strains were found sensitive to endocytosis of macrophages. Mitogen-activated protein kinase (MAPK) signaling pathway of macrophages was significantly activated by K7R^R^ and GT-deficient strains comparing with that of K7. Interestingly, in the presence of macromolecular CPS residues (>250 KD), K7(Δ*GT-1*) and K7(Δ*wcaJ*) could still be bounded by GH-K3, though with a modest adsorption efficiency, and showed minor virulence, suggesting that the CPS residues accumulated upon deletion of *GT-1* or *wcaJ* did retain phage binding sites as well maintain mild virulence. In brief, our study defines, for the first time, the potential roles of GT-1, GT-2, and WcaJ in *K. pneumoniae* in bacterial virulence and generation of rough-type mutation under the pressure of bacteriophage.

## Introduction

As important nosocomial and community-acquired opportunistic pathogens, *Klebsiella pneumoniae* (*K. pneumoniae*) spp. cause various infections, such as pneumonia, liver abscess, urinary tract infections, and complicated bacteremia (Podschun and Ullmann, 1998). In recent years, with the prevalence of antibiotic-resistant strains, traditional antibiotic therapies for infections caused by multidrug-resistant (MDR) *K. pneumoniae* has been limited (Munoz-Price et al., 2013; Robilotti and Deresinski, 2014; Humphries and Hemarajata, 2017). Therefore, due to the urgent need for alternative of antibiotic therapies, phage therapies have regained attention (Nobrega et al., 2015). However, *K. pneumoniae* strains are highly inclined to form phage-resistant mutants during phage treatment, which greatly limits the phage therapies (Labrie et al., 2010).

With sophisticated mechanisms, anti-phage mutations of bacteria usually occur at various stages of phage infection (Labrie et al., 2010). Especially, the abortion of adsorption is the most common bacteriophage resistance mechanism (Silva et al., 2016). Similar to other Gram-negative bacteria, the first and second phage adsorption receptors based on polysaccharides and outer membrane proteins of *K. pneumoniae* have been described (Cai et al., 2018). During the interaction of phage and host strain, majority of the anti-phage mutant colonies became small-rough type due to loss of cell-surface polysaccharides. Polysaccharides of various Gram-negative bacteria mainly comprised of lipopolysaccharide (LPS) (O antigen) and capsular polysaccharide (CPS) (K antigen) on the cell surface, which are directly regulated by *lps* and *cps* gene clusters, respectively (Whitfield, 2006). The LPS or CPS have been recognized as adsorption receptors for many phages (Pickard et al., 2010; Kiljunen et al., 2011; Sorensen et al., 2011). With mutation in LPS or CPS synthetic genes, O antigen or K antigen of phage-resistant strains is generally lost resulting phenotype with comparatively smaller colony and much lower phage adsorption efficiency than the parental wild type strains (Le et al., 2014; Li et al., 2018). Both O antigen and K antigen are involved in phage adsorption on *K. pneumoniae* (Thurow et al., 1975; Tomás et al., 1987), but which component serves as primary phage adsorption receptor is still inconclusive.

Because of enhancing bacterial adherence and protecting the strains from complement-mediated killing through opsonophagocytosis, LPS and CPS have also been widely recognized as the virulence factors (Alberti et al., 1996; Mushtaq et al., 2005). In our previous study, the virulence of *K. pneumoniae* K7-derived phage resistant strains were dramatically weaker than the wild-type strain (Gu et al., 2012), indicating that the variation of virulence factor might have occurred. With at least 79 serotypes have been recognized (Hsu et al., 2013; Pan et al., 2015a), virulence factor coding genes of *K. pneumoniae* are highly diverse. In addition to polysaccharides-related gene clusters, bacterial virulence is also regulated by transcriptional factors. It had been reported that the regulator of mucoid phenotype A (RmpA) plays as an important regulatory factor for maintaining mucoidy and pathogenicity of *K. pneumoniae* (Lai et al., 2003; Hsu et al., 2011). Rcs phosphorelay system also serves as modulators for polysaccharide synthesis-associated gene clusters (Su et al., 2018). Besides, the regulation of CPS synthesis by RcsB depends on the auxiliary role of RmpA (Cheng et al., 2010).

In the present work, the mechanisms of anti-phage mutation and virulence reduction of rough-type phage-resistant *K. pneumoniae* were revealed. By quantitative analysis with liquid chromatography-tandem mass spectrometry (LC-MS-MS), the abundance of three glycosyltransferases, GT-1, GT-2, and WcaJ was drastically altered in K7R^R^ compared with the wild-type K7. Then the potential roles of three glycosyltransferases in bacterial virulence reduction and phage-resistance were studied.

## Materials and Methods

### Ethics Statement

C57BL/6J mice (Female, 18–20 g) were purchased from Liaoning Changsheng Biotechnology Co. LTD (Benxi, Liaoning, China). All animal managements and experiments were strictly abided by the Regulations for the Administration of Affairs Concerning Experimental Animals approved by the State Council of the People’s Republic of China (1.11.1988) and approved by the Animal Welfare and Research Ethics Committee at Jilin University.

### Bacterial Strains and Phage

Phage GH-K3 and its host strain *K. pneumoniae* K7 were previously isolated and maintained in our laboratory (Gu et al., 2012). A rough-type, phage-resistant *K. pneumoniae* mutant strain was isolated and named as K7R^R^ in a previous study (Cai et al., 2018).

### Phage Adsorption Assay

*Klebsiella pneumoniae* strains were cultured until late-exponential phase (2 × 10^9^ CFU/ml) and mixed with phages at the quantity ratio of 100:1 (bacteria: phage). The mixtures were incubated for 10 min at 37°C with shaking and then centrifuged at 4°C (13,000 × *g*, 5 min). After the supernatants were filtered with 0.22 μm filters (Millipore, Billerica, MA, United States), the phage particles remaining in supernatants were measured by double-plate assay in triplicate and the phage adsorption efficiency were calculated as previously described (Le et al., 2013; Cai et al., 2018).

### LC-MS-MS Analyses

K7 and K7R^R^ samples (three biological replicates) used for proteomic analyses were prepared as previously described (Hu et al., 2014; Liu et al., 2015). LC-MS-MS analyses were executed using a linear ion trap mass spectrometer (LTQ Velos Pro, Thermo Scientific, San Jose, CA, United States) equipped with nanoflow reversed-phase liquid chromatography (EASY-nLC 1000; Thermo Scientific, San Jose, CA, United States) at Peking University. Instrument parameter settings, sample loadings and subsequent data analyses were referred to our previous study (Cai et al., 2018). Raw MS files were searched with Mascot (version 2.3.02; Matrix Science Inc.) against annotated protein sequences of *K. pneumoniae* K7 (Genbank accession number: NKQH00000000). In order to classify differentially expressed protein data visually, a heat map analysis was performed by Heml 1.0 (Deng et al., 2014). Nomenclature of the heat map numbering scale and COG analysis were both processed by STRING (Szklarczyk et al., 2017).

### Scanning Electron Microscopy

*Klebsiella pneumoniae* strains were grown to exponential phase (OD_600_ = 0.4–0.6) and then washed three times with sterile phosphate-buffered saline (PBS). After immobilized with 4% glutaraldehyde overnight, the strains were dehydrated by ethanol with different concentrations (20, 50, 70, 90, and 100%) and subsequently freeze-dried on cover glasses. Finally, bacterial surface morphology was observed by scanning electron microscopy (SEM) (Hitachi S-3400N, Hitachi High-Technologies Europe GmbH, Krefeld, Germany).

### Construction of Gene Deleted Mutant Strains

K7(Δ*GT-1*), K7(Δ*GT-2*), and K7(Δ*wcaJ*) were constructed as previously described (Guo et al., 2014). Briefly, homologous recombination arms of *GT-1*, *GT-2*, and *wcaJ* obtained by overlap extension PCR were amplified and cloned into pUC19 cloning vector. After digestion with *Sal* I, these sequences used for target gene replacement were individually ligated to pCVD442(Km^r^). Followed by electrotransformation into *Escherichia coli* SM10 λpir, the plasmid bearing strains were mixed with the recipient strain- *K. pneumoniae* K7 (1:1 *v*/*v*). The mixed bacteria were then centrifuged at 6,000 × *g* for 10 min and suspended with LB medium on a sterile membrane (Millipore, Billerica, MA, United States) at 30°C overnight. The mixed bacteria attached to the membrane were eluted with sterile saline and plated onto LB plates containing 30 μg/ml kanamycin and 100 μg/ml ampicillin. After 12 h of culture, the putative transconjugants of K7(Δ*GT-1*), K7(Δ*GT-2*), and K7(Δ*wcaJ*) were screened and verified by PCR. All primers used in this study are listed in Supplementary Table S1. The strains and plasmids used in this work are listed in Supplementary Table S2.

### Polysaccharide Extraction and Electrophoresis

For polysaccharide analyses, *K. pneumoniae* strains were inoculated in LB medium and cultured overnight. Exopolysaccharide (EPS), including both CPS and LPS, were isolated from equal amounts of *K. pneumoniae* strains (1.0 × 10^9^ CFU) by hot water-phenol extraction method as previously reported (Chuang et al., 2006). The polysaccharide solution obtained by chloroform extraction was further purified according to the method as previously described (Majkowska-Skrobek et al., 2016). Briefly, the nucleic acids and proteins in supernatant were precipitated by the addition of trichloroacetic acid (TCA; 20% *w*/*v*), and the solution was then centrifuged to discard the precipitate (16,000 × *g*, 1 h, 4°C). Next, the supernatant was passed through 3 volumes of 96% cold ethanol and placed at -20°C for 24 h, then the precipitate was collected by centrifugation (16,000 × *g*, 1 h, 4°C) and resuspended in deionized water. After removal of the LPS component by ultracentrifugation (100,000 × *g*, 20 h, 4°C), the crude CPS was dialyzed against an excess deionized water using a SERVAPOR^®^ dialysis tubing (Serva, Heidelberg, Germany) with a molecular weight cut-off (MWCO) of 12–14 kDa. After removing low molecular weight impurities for 24 h (4°C), the purified CPS was lyophilized and weighed. The purity of CPS was verified as previously described (Cano et al., 2009).

Lipopolysaccharide of these strains was purified with LPS extraction kit (17141, iNtRON Biotechnology, Korea) according to the manufacturer’s instructions. After separated by 12%-sodium dodecylsulfate-polyacrylamide gel electrophoresis (SDS-PAGE), CPS and LPS were visualized by alcian blue staining and silver staining, respectively (Fomsgaard et al., 1990; Moller et al., 1993). Additionally, polysaccharide quantification was performed by using BCPS-PURPALD ASSAY KIT as per manufacturer’s instruction (GenMed Scientifics, Shanghai, China).

### Quantitative Analyses of mRNA Level

The strain cultures were pelleted and washed twice with PBS. Then the total RNA was extracted using the Bacterial RNA Kit (Omega Bio-Tek Inc., Norcross, GA, United States). Less than 1 μg of total RNA was converted to cDNA using the PrimeScript RT reagent Kit with gDNA Eraser (Takara, Dalian, China). Subsequently, qRT-PCR was carried out utilizing SYBR^®^ Premix Ex Taq^TM^ II (Tli RNaseH Plus) (Takara, Dalian, China). The primers were listed in Supplementary Table S1. Two-step programs were run on Applied Biosystems^®^ 7500 Real-Time PCR System (Thermo Fisher, Germany) and all reactions were run in triplicate. Reactions were performed as follows: initial denaturation at 95°C for 30 s, followed by 40 cycles of 5 s at 95°C and 30 s at 60°C. Lastly, the temperature was increased from 55 to 95°C at a rate of 0.2°C/s to establish a melting curve. Gene expression levels were calculated by the delta CT method as follows: fold-change = 2^-ΔΔCt^, where ΔCt = Ct (specific transcript)-Ct (housekeeping transcript) and ΔΔCt = ΔCt (treatment) – ΔCt (control).

### Determination of Virulence of Wild-Type and Mutant *K. pneumoniae* Strains

The virulence of wild-type and mutant *K. pneumoniae* strains were determined according to a previous study with some modifications (Xia et al., 2016). The mice were anesthetized intraperitoneally with ketamine (100 mg/kg) and xylazine (10 mg/kg), and were inoculated intranasally with different doses of *K. pneumoniae* K7, K7R^R^, K7(Δ*GT-1*), K7(Δ*GT-2*), and K7(Δ*wcaJ*) suspensions (5 × 10^5^, 5 × 10^6^, 5 × 10^7^, 5 × 10^8^, 1 × 10^9^, or 5 × 10^9^ CFU/mouse) to determine the minimum dose that triggered 100% mortality over a 7-day follow-up period (minimum lethal dose [MLD]). In addition, in order to detect the survival rate of the same infection level, mice were challenged intranasally with 1 × 10^7^ CFU/mouse [2 × MLD of K7] of *K. pneumoniae* K7, K7R^R^, K7(Δ*GT-1*), K7(Δ*GT-2*), and K7(Δ*wcaJ*) (*n* = 10 in each group). The number of dead mice was recorded on daily basis. Finally, all the surviving mice were euthanized by intravenous injection of Fatal Plus (pentobarbital sodium) (100 mg/kg).

From each treatment group, three mice were randomly selected at 48 h post infection and were euthanized with Fatal Plus (pentobarbital sodium) (100 mg/kg). After carefully removed from thoracic cavity and photographed, the left and right lung tissues were separated and weighed immediately. The left lungs were fixed in 4% formalin and then embedded in paraffin. Then hematoxylin and eosin (H&E) staining was performed (Feldman and Wolfe, 2014). Briefly, paraffin sections were re-hydrated and stained with hematoxylin for 2–4 min. After washing with water, they were subsequently stained with eosin for 0.5–1 min. Followed by dehydration treatment, the sections were mounted with xylene based mounting medium. After that, histopathology analyses were performed by microscopy. To measure the level of cytokines, the right lung lobes were lysed using Tissue Extraction Reagent (Invitrogen, Waltham, MA, United States) according to the manufacturer’s instructions followed by homogenition with a probe sonicator (Branson Ultrasonics, Danbury, CT, United States). The supernatants from the homogenates were obtained by centrifugation (5,000 × *g*, 10 min) at 4°C. Concentrations of interleukin-1β (IL-1β), interleukin-6 (IL-6), tumor necrosis factor-alpha (TNF-α), and interferon gamma (IFN-γ) in supernatants were detected using enzyme-linked immunosorbent assay (ELISA) kits (eBioscience, San Diego, CA, United States) according to the manufacturer’s instructions.

### Macrophage Phagocytosis and Cell Cytotoxicity Assay

The RAW264.7 cell line (ATCC HTB37) was purchased from the American Type Culture Collection (ATCC, Rockville, MD, United States) and maintained in Dulbecco’s modified Eagle’s medium (DMEM) (10% FBS, 100 U/ml penicillin and 100 mg/ml streptomycin) (all from Gibco BRL, Gaithersburg, MD, United States) at 37°C and 5% CO_2_.

*Klebsiella pneumoniae* K7, K7R^R^, K7(Δ*GT-1*), K7(Δ*GT-2*), and K7(Δ*wcaJ*) were stained with SYTO^TM^ 9 Green Fluorescent Nucleic Acid Stain (catalog number: S34854, Invitrogen, Carlsbad, CA, United States) according to the manufacturer’s instructions and washed two times with PBS. RAW264.7 cells were seeded on 35 mm glass bottom culture dishes (801001, NEST, Wuxi, China). When adherent cells reached a density of 5 × 10^5^ cells per well, the cell culture medium was changed to fresh DMEM without adding antibiotics and FBS, and the stained *K. pneumoniae* strains were added to the supernatants at a multiplicity of infection (MOI) of 10:1. After co-incubated with strains at 37°C for 2 h, the cells were washed three times and treated with gentamicin (200 μg/ml) for 1 h to kill the extracellular bacteria. The cells were then fixed in 4% paraformaldehyde (Sigma-Aldrich, St. Louis, MO, United States) for 15 min. Followed by permeabilization with 0.5% Triton X-100 (Sigma-Aldrich, St. Louis, MO, United States), cytoskeleton was stained with Phalloidin-iFluor 555 (ab176756; Abcam, Cambridge, MA, United States) (1:200 in 1% BSA) for 30 min. After two washes, nucleus was stained with Hoechst 33342 (Invitrogen, Waltham, MA, United States) (1:200) for 10 min. Endocytosis of cells incubated with *K. pneumoniae* was detected with laser scanning confocal microscopy (LSM710; Carl Zeiss, Germany).

Quantitative detection of intracellular bacterial loads was performed as previously described (Shi et al., 2016). The RAW264.7 cells were cultured in 12-well plates. When adherent cells reached a density of 5 × 10^5^ cells per well, the *K. pneumoniae* strains were added to the supernatants (without antibiotics and FBS) at an MOI of 10:1 and coincubated for 2 h at 37°C. After gentamicin treated and washing three times with sterile PBS, the cells were lysed in 1% Triton X-100 immediately. Finally, the bacterial loads in each cell samples were measured by plating onto LB agar with multiple serial dilutions.

Meanwhile, cytotoxicity of the cells was measured in a 96-well plate by using CellTiter 96^®^ AQ_ueous_ One Solution Reagent (MTS) (Promega, Madison, WI, United States) according to the manufacturer’s instructions after incubation with purified CPS from K7 (10 μg) and different *K. pneumoniae* for 2 or 12 h. Cells without any treatment were served as controls. Cell viability was calculated as: (OD_490 sample_- OD_490 blank_)/(OD_490 control_-OD_490 blank_) × 100. In addition, the multiplication efficacy of different bacteria in DMEM was measured by bacterial count.

### Western Blot Analyses

To detected the expression levels of GT-1, GT-2, and WcaJ in different *K. pneumoniae* strains, polyclonal antibodies targeting the peptide antigens (Supplementary Table S3) of glucosyltransferases were prepared by PolyExpress^TM^ at GenScript Biological Technology Co., Ltd (Nanjing, China). To measure the signaling pathways of RAW264.7 cells, phospho-p38 monoclonal antibody (4511) and phospho-NF-κB p65 monoclonal antibody (4025) were purchased from Cell Signaling Technology (Danvers, MA, United States). Total proteins in RAW264.7 cells were extracted by T-PER^TM^ Tissue Protein Extraction Reagent and quantified by Pierce^TM^ BCA Protein Assay Kit according to the manufacturer’s instructions (both from Thermo Scientific, Rockford, IL, United States). Identical amounts of each protein sample were separated by 12% SDS-PAGE prior to transfer to polyvinylidene difluoride (PVDF) membranes (Millipore, Billerica, MA, United States). Immunoblot membranes were sequentially incubated with primary antibodies [anti-GT-1 (1: 1000), anti-GT-2 (1: 1000), anti-WcaJ (1: 2000), anti-OmpC (1: 1000), anti-DnaK (1: 2000), anti-pp38 (1: 1000), anti-pp65 (1: 1000), or anti-β-actin (1:1000)] and labeled with horseradish peroxidase (HRP)-conjugated goat anti-rabbit antibody (ab205718; Abcam, Cambridge, MA, United States) (1: 5000). Immunoblotting was performed using Immobilon^TM^ Western Chemiluminescent HRP Substrate (Millipore, Billerica, MA, United States) and detected with a chemiluminescence imaging system (Tanon 5200, Shanghai, China).

### Statistical Analysis

Survival curve analyses were performed using log-rank (Mantel–Cox) test. While other statistical data presented in this study were processed by One-way or Two-way analysis of variance (ANOVA) or Student’s *t-*tests. All charts are generated by GraphPad Prism 6 (GraphPad Software, Inc., San Diego, CA, United States). Standard error of the mean was represented by error bars. The data differences were considered statistically significant at *P*-values < 0.05.

## Results

### K7R^R^ Showed Low Virulence in Mice Model

**FIGURE 1:**
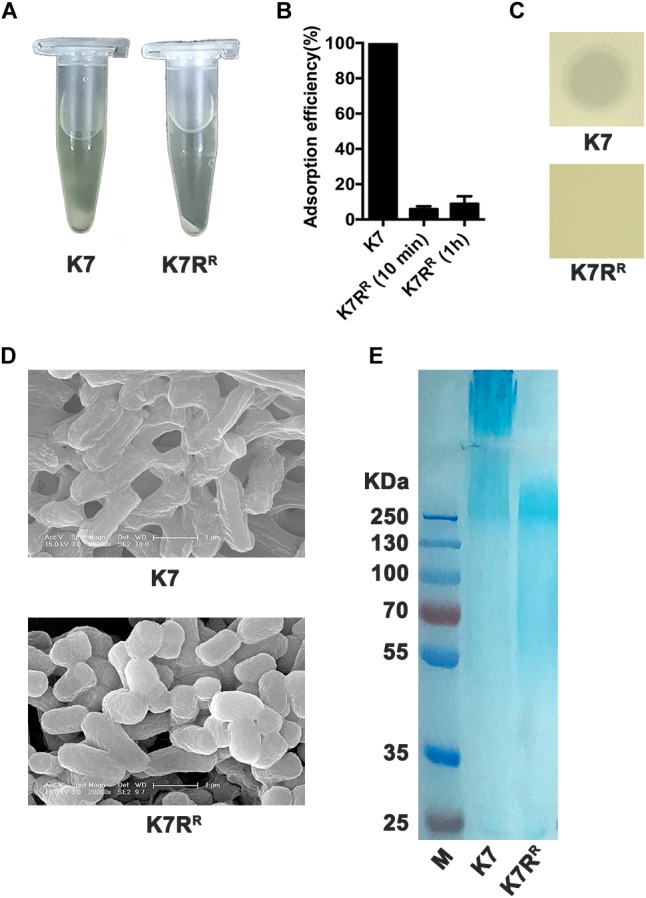
Characteristics of *K. pneumoniae* K7 and K7R^R^. **(A)** Centrifugation analysis of *K. pneumoniae* K7 and K7R^R^. The cultures were centrifuged at 10,000 × *g* for 5 min at 4°C. **(B)** Adsorption efficiency of GH-K3 binding to *K. pneumoniae* K7 and K7R^R^. Data represent the mean ± SEM of triplicate experiments. **(C)** Spot assays for GH-K3 on *K. pneumoniae* K7 and K7R^R^. Five μl of phage solutions were spotted onto freshly lawns of *K. pneumoniae* K7 and K7R^R^ for 6 h at 37°C before observation. **(D)** SEM analyses of the surface morphology of *K. pneumoniae* K7 and K7R^R^. Scale bars represent 1 μm. **(E)** CPS samples were extracted from equal amounts of *K. pneumoniae* strains (1.0 × 10^9^ CFU). After separated by 12% SDS-PAGE, CPS phenotypes of K7 and K7R^R^ were visualized by alcian blue staining.

GH-K3 could not form plaques on *K. pneumoniae* K7R^R^ like other phage-resistant strains. Compared with *K. pneumoniae* K7, the precipitate of K7R^R^ was compact after centrifugation (10,000 × *g*, 5 min) (Figure 1A). Phage GH-K3 had been confirmed to possess an extremely low adsorption efficiency on *K. pneumoniae* K7R^R^ (3.9%) (Cai et al., 2018). We found that the adsorption efficiency of this phage showed very little improvement after prolonging the infection time to 1 h (8.9%) (Figure 1B). Besides, GH-K3 could not form spots on a lawn of K7R^R^ (Figure 1C). SEM analyses also showed that the boundaries of K7R^R^ were clearer than those of K7 strains (Figure 1D). Additionally, compared with the high- molecular weight CPS in K7, the molecular weight distribution of the polysaccharide residues in K7R^R^ was very wide, and some oligosaccharides (approximately 50 KD) were also included (Figure 1E), suggesting that the capsule synthesis was possibly interrupted on the cell surface of K7R^R^. While, with the increased contents, the LPS components in K7R^R^ were not affected by the loss of CPS (Supplementary Figure S1A).

**FIGURE 2:**
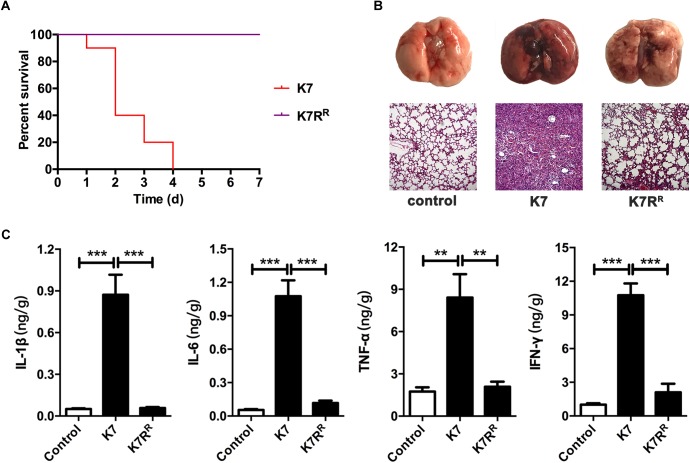
Virulence evaluation of *K. pneumoniae* K7 and K7R^R^ *in vivo*. All the mice were challenged intranasally with 1 × 10^7^ CFU/mouse of *K. pneumoniae* K7 and K7R^R^. **(A)** Survival rates of mice challenged with *K. pneumoniae* K7 and K7R^R^. Each group contained ten mice. Statistical analysis was performed using the Kaplan–Meier method [*P* < 0.0001, log-rank (Mantel–Cox) test]. **(B)** Pathological observation. At 48 h after infection, the lungs from mice challenged with *K. pneumoniae* K7 and K7R^R^ were carefully removed and photographed after euthanasia. Lung tissue sections were stained with hematoxylin and eosin (H&E) and then observed in a microscope at a magnification 100×. Lung tissues of healthy mice were served as controls. **(C)** The levels of cytokines. The levels of IL-1β, IL-6, TNF-α, and IFN-γ in lung tissue homogenates of mice infected by *K. pneumoniae* K7 and K7R^R^ were determined. Lung tissue homogenates of healthy mice were served as controls. ^∗∗^ and ^∗∗∗^ represent significant differences at *P* < 0.01 and *P* < 0.001, respectively. Data represent the mean ± SEM of triplicate experiments.

As a hypervirulent *K. pneumoniae* strain with K2 serotype (Cai et al., 2018), the minimum lethal dose (MLD) of intranasal inoculation of K7 was determined as 5.0 × 10^6^ CFU/mouse. However, the rough-type variant strain K7R^R^ showed much weaker virulence with an MLD of 1.0 × 10^9^ CFU/mouse. K7 infection (1.0 × 10^7^ CFU/mouse, intranasally) significantly induced the up-regulation of pro-inflammatory cytokines in mice. The mice infected with K7 were all died within 96 h and the alveolar structures of their lungs were found almost completely lost. By contrast, mice challenged with K7R^R^ (same dose as K7) were all survived and showed much reduced damage to lungs. In addition, no significant difference was detected in the levels of cytokines including IL-1β, IL-6, TNF-α, and IFN-γ comparing with those of healthy mice at 48 h after infection (Figure 2).

### Differential Protein Expression Between K7 and K7R^R^

**FIGURE 3:**
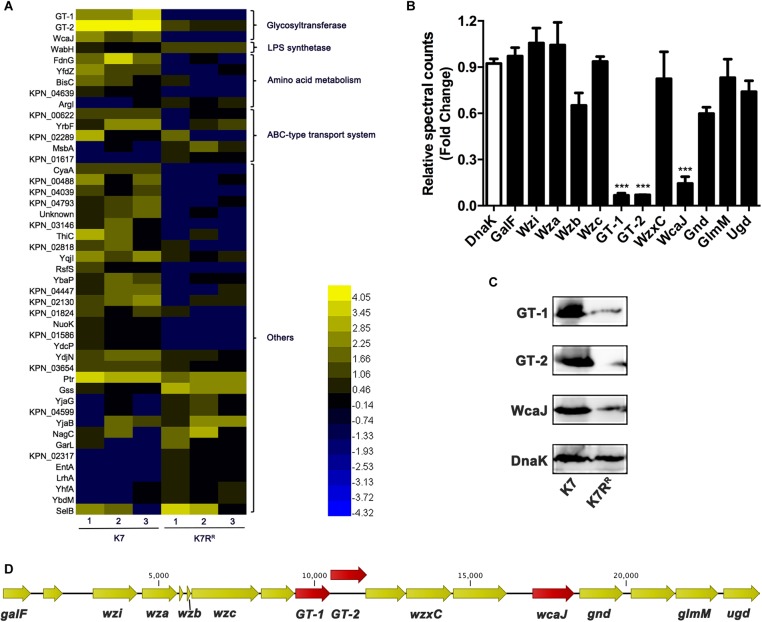
The differentially expressed proteins between *K. pneumoniae* K7 and K7R^R^. **(A)** Heat-map analysis of differentially expressed proteins between *K. pneumoniae* K7 and K7R^R^. The heat-map was generated by Heml 1.0. Lanes 1–3 represent three biological replicates at each sampling time. Protein functional classification was performed by STRING, version 10.5. **(B)** Spectral count fold changes. The relative spectral counts of proteins encoded by *cps* gene cluster between *K. pneumoniae* K7 and K7R^R^ were analyzed. DnaK served as a loading control. Relative spectral count fold changes = (spectral counts of K7R^R^)/(spectral counts of K7). ^∗∗∗^represents significant differences at *P* < 0.001. Data represent the mean ± SEM of triplicate biological experiments at each sampling time. **(C)** Western blot analyses of GT-1, GT-2, and WcaJ. Expression levels of GT-1, GT-2, and WcaJ in *K. pneumoniae* K7 and K7R^R^ were detected. Expression of DnaK was detected as a control. **(D)** Schematic of *cps* gene cluster of *K. pneumoniae* K7 indicated by arrows with different colors. The arrow represents the direction of transcription.

About 3100 proteins of K7 and K7R^R^ were compared after analyzed by LC-MS-MS. A total of 46 proteins of K7R^R^ were identified as differential proteins, including 16 up-regulated proteins and 30 down-regulated proteins (Figure 3A). Of these proteins, three glycosyltransferases in K7R^R^ were drastically repressed in abundance, including GT-1 (∼16.7-fold), GT-2 (∼14.2-fold), and WcaJ (∼8.1-fold) (Figure 3A,B), which belonged to Pfam family Glycos_transf_1 (PF00534), Pfam Family: Glyco_trans_1_4 (PF13692) and Pfam Family: Bac_transf (PF02397), respectively. Western blot and qPCR analyses further confirmed the down-regulation of these three proteins at the protein and mRNA levels, respectively (Figure 3C and Supplementary Figure S2A). As a colanic acid biosynthesis UDP-glucose lipid carrier transferase, WcaJ is mainly found in the strains with K1, K2, K5, K14, or K64 serotype CPS, serving as an important basis for the capsule classification of *K. pneumoniae* (Shu et al., 2009; Pan et al., 2015b). Besides *WcaJ*, *GT-1*, and *GT-2* are also located in a *cps* gene cluster consisting of 18 ORFs (between *galF* and *ugd*) (Figure 3D). So far, the actual roles of GT-1 and GT-2 in *K. pneumoniae* have not been reported. Surprisingly, besides *GT-1*, *GT-2*, and *wcaJ*, *K. pneumoniae* K7R^R^ has no genetic mutation at the genome level compared to K7.

Except for GT-1, GT-2, and WcaJ, the expression levels of other encoded proteins in the *cps* cluster showed no obviously change in K7R^R^ (Figure 3B). In addition, the expression levels of LPS synthesis-related proteins in K7R^R^ have also been changed. WabH, a lipopolysaccharide core biosynthesis glycosyl transferase, was upregulated ∼2.5-fold in abundance. ATP-binding-cassette (ABC) transporters typically drive uptake or secretion of substrates at the expense of ATP hydrolysis to ensure energy supply during sugar chain polymerization (Whitfield, 2006). Some of them were also moderately altered for ∼2.0-fold to 3.0-fold in expression levels, including three down-regulated proteins (KPN_00622, YrbF and KPN_02289) and two up-regulated proteins (MsbA and KPN_01617) (Figure 3A).

### Deletions of GT-1, GT-2, or WcaJ Created Resistant Phenotype to Phage GH-K3

**FIGURE 4:**
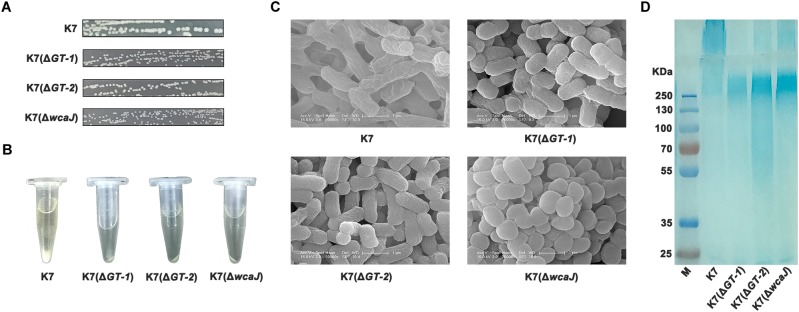
Characteristics of *K. pneumoniae* K7(Δ*GT-1*), K7(Δ*GT-2*), and K7(Δ*wcaJ*). **(A)** Colony morphologies of *K. pneumoniae* K7(Δ*GT-1*), K7(Δ*GT-2*), and K7(Δ*wcaJ*). Colonies of K7(Δ*GT-1*), K7(Δ*GT-2*), and K7(Δ*wcaJ*) were cultured on LB plates at 37°C for 12 h after streak plating. The three mutant strains all form rough-type colonies. K7 was used as a control. **(B)** Centrifugation analysis of *K. pneumoniae* K7(Δ*GT-1*), K7(Δ*GT-2*), and K7(Δ*wcaJ*). The cultures were centrifuged at 10,000 × *g* for 5 min. K7 was used as a control. **(C)** SEM analyses of the surface morphology of *K. pneumoniae* K7(Δ*GT-1*), K7(Δ*GT-2*), and K7(Δ*wcaJ*). Scale bars represent 1 μm. K7 was used as a control. **(D)** CPS samples were extracted from equal amounts of *K. pneumoniae* strains (1.0 × 10^9^ CFU). After separated by 12% SDS-PAGE, CPS phenotypes of K7(Δ*GT-1*), K7(Δ*GT-2*), and K7(Δ*wcaJ*) were visualized by alcian blue staining. CPS of K7 was used as a control.

To identify the effects of GT-1, GT-2, and WcaJ in K7 on phage sensitivity, single gene deletion of *GT-1*, *GT-2*, and *wcaJ*, respectively, was performed in K7. As shown in Figure 4A, all the three GT-deficient strains K7(Δ*GT-1*), K7(Δ*GT-2*), and K7(Δ*wcaJ*) appeared small and rough type in colony morphology. Besides, compared with K7, the three mutant strains tend to form compact precipitation as K7R^R^ during centrifugation and mucoid polysaccharide capsules on the cell surface were almost disappeared (Figure 4B,C). Interestingly, K7(Δ*wcaJ*) resembled cocci unlike rod shape of K7, indicating that WcaJ may play a critical role in maintenance of bacterial morphology.

By alcian blue staining, the molecular weights of polysaccharides in K7(Δ*GT-1*) and K7(Δ*wcaJ*) were found above 250 KD, but much lower than the CPS polymer of K7. While, like K7R^R^, molecular weight of polysaccharide residues in K7(Δ*GT-2*) was widely distributed and some oligosaccharides (approximately 50 kD) were also detected. (Figure 4D), indicating that sugar chain polymerization in K7(Δ*GT-2*) might be further hindered compared with K7(Δ*GT-1*) and K7(Δ*wcaJ*). In addition, the composition of LPS in three GT-deficient strains was not changed and the production was obviously higher than that of K7 (Supplementary Figure S1B).

By western blot and qPCR analyses, the regulatory relationships among GT-1, GT-2, and WcaJ has been revealed. In both mRNA and protein levels, WcaJ was also down-regulated when the *GT-1* was deleted. The expression level of GT-2 was severely affected when *wcaJ* was deleted. However, the deletion of *GT-2* did not have a significant effect on the expression of GT-1 and WcaJ (Supplementary Figures S2B, S3), indicating that this glucosyltransferase may play an independent role in the assembly of sugar chains.

**FIGURE 5:**
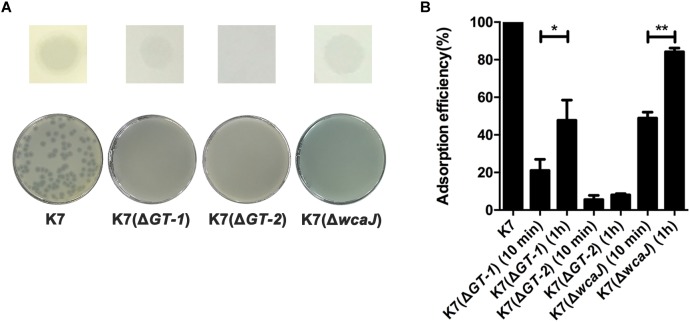
Sensitivity of *K. pneumoniae* K7(Δ*GT-1*), K7(Δ*GT-2*), and K7(Δ*wcaJ*) to GH-K3. **(A)** Spot and plaque forming assays. Five μl of GH-K3 solutions were spotted onto freshly seeded lawns of *K. pneumoniae* K7(Δ*GT-1*), K7(Δ*GT-2*), and K7(Δ*wcaJ*) for 6 h at 37°C before observation (top). Meanwhile, the sensitivity of *K. pneumoniae* K7(Δ*GT-1*), K7(Δ*GT-2*), and K7(Δ*wcaJ*) to GH-K3 were determined by plaque assays (bottom). K7 was used as a control. **(B)** Adsorption efficiency. The adsorption efficiency of GH-K3 binding to *K. pneumoniae* K7(Δ*GT-1*), K7(Δ*GT-2*), and K7(Δ*wcaJ*) were determined. ^∗^ and ^∗∗^ significant differences at *P* < 0.05 and *P* < 0.01, respectively. Data represent the mean ± SEM of triplicate experiments.

The sensitivity of K7(Δ*GT-1*), K7(Δ*GT-2*), and K7(Δ*wcaJ*) to phage GH-K3 was further measured. GH-K3 was unable to form plaques on any of the deficient strains K7(Δ*GT-1*), K7(Δ*GT-2*), and K7(Δ*wcaJ*). However, the phage could form spots on lawns of both K7(Δ*GT-1*) and K7(Δ*wcaJ*) (Figure 5A), suggesting that some phage-encoded proteins (such as lysin or depolymerase) of GH-K3 may recognize the macromolecular CPS residues of these two mutant strains. In spite of the adsorption efficiencies of GH-K3 on K7(Δ*GT-1*), K7(Δ*GT-2*), and K7(Δ*wcaJ*) were all markedly reduced (down to 21.1, 5.5, and 48.9%, respectively), the adsorption efficiencies of phage GH-K3 on both K7(Δ*GT-1*) and K7(Δ*wcaJ*) have a significant improvement after prolonging the adsorption time (1 h), with the adsorption efficiencies of 47.8 and 84.2%, respectively. However, the adsorption efficiency of GH-K3 on K7(Δ*GT-2*) did not change significantly with the extension of time, only reached 8.0% finally (Figure 5B).

### Deletions of GT-1, GT-2, or WcaJ Resulted in the Decline of Virulence

**FIGURE 6:**
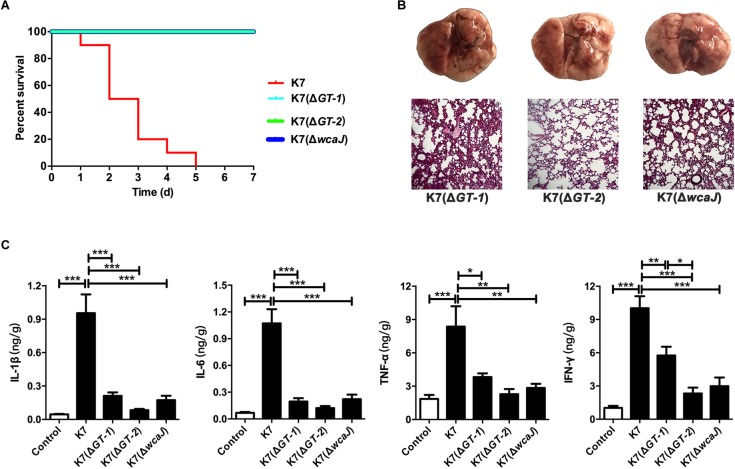
Virulence evaluation of *K. pneumoniae* K7(Δ*GT-1*), K7(Δ*GT-2*), and K7(Δ*wcaJ*). All the mice were challenged intranasally with 1 × 10^7^ CFU/mouse of *K. pneumoniae* K7, K7(Δ*GT-1*), K7(Δ*GT-2*), and K7(Δ*wcaJ*). **(A)** Survival rates. Survival rates of mice challenged with *K. pneumoniae* K7(Δ*GT-1*), K7(Δ*GT-2*), and K7(Δ*wcaJ*) were determined. Each group contained ten mice. Statistical analysis was performed using the Kaplan–Meier method by [*P* < 0.0001, log-rank (Mantel-Cox) test]. **(B)** Pathological observation. At 48 h after infection, the lungs of mice challenged with *K. pneumoniae* K7(Δ*GT-1*), K7(Δ*GT-2*), and K7(Δ*wcaJ*) were carefully removed and photographed after euthanasia. Lung tissue sections were stained with H&E and then observed in a microscope at a magnification 100×. Lung tissues of healthy mice were served as controls. **(C)** The levels of four cytokines. The levels of IL-1β, IL-6, TNF-α, and IFN-γ in lung tissue homogenates of mice infected by *K. pneumoniae* K7(Δ*GT-1*), K7(Δ*GT-2*), and K7(Δ*wcaJ*) were determined. Lung tissue homogenates of healthy mice were served as controls. ^∗^, ^∗∗^, and ^∗∗∗^ represent significant differences at *P* < 0.05, *P* < 0.01, and *P* < 0.001, respectively. Data represent the mean ± SEM of triplicate experiments.

For C57BL/6J mice, the MLDs for intranasal inoculation of K7(Δ*GT-1*), K7(Δ*GT-2*), and K7(Δ*wcaJ*) are 5.0 × 10^8^ CFU/mouse, 1.0 × 10^9^ CFU/mouse, and 5.0 × 10^8^ CFU/mouse, respectively. According to the results of our 7-day monitoring, mice in all three groups survived after undergoing transient respiratory symptoms after administration with different mutant strains at a dose of 1.0 × 10^7^ CFU/mouse (Figure 6A). Like K7R^R^-infected mice, the lungs of K7(Δ*GT-1*), K7(Δ*GT-2*), and K7(Δ*wcaJ*)-challenged mice showed mild hyperemia, but the texture was still tough. The histopathological changes of lung tissues in K7(Δ*GT-1*) and K7(Δ*wcaJ*) infection groups were very similar. Although the alveolar walls had partial telangiectasia with a small amount of collapse, most of the alveolar structures maintained their normal morphology. While the lung tissues of mice infected with K7(Δ*GT-2*) had a better condition, similar to that of healthy mice (Figure 6B). In addition, cytokine levels in the K7(Δ*GT-2*) group was close to the level of healthy mice at 48 h post infection, while K7(Δ*GT-1*) and K7(Δ*wcaJ*) group were slightly higher than that of K7(Δ*GT-2*) group (Figure 6C).

### GTs-Deficient *K. pneumoniae* Strains Promote Endocytosis and Activation Effects of Macrophages and Show Weaker Cytotoxicity

**FIGURE 7:**
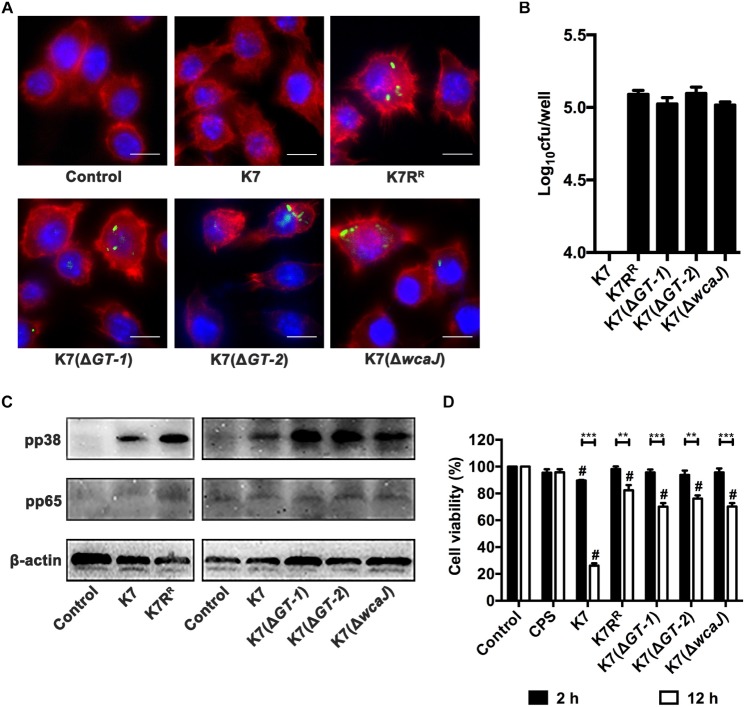
Endocytosis, activation and cytotoxicity of RAW264.7 cells after incubation with different *K. pneumoniae* strains. **(A)** Immunofluorescence analysis of endocytosis effects of RAW264.7 cells after incubation with different *K. pneumoniae* strains. *K. pneumoniae* K7, K7R^R^, K7(Δ*GT-1*), K7(Δ*GT-2*), and K7(Δ*wcaJ*) were stained with SYTO^TM^ 9 Green Fluorescent Nucleic Acid Stain. The *K. pneumoniae* strains were then co-incubated with RAW264.7 cells in antibiotic-free supernatants. Cytoskeleton and nuclei were stained with Phalloidin-iFluor 555 and Hoechst 33342, respectively. The endocytosis of these *K. pneumoniae* strains in RAW264.7 cells was determined with a laser scanning confocal microscope and then observed at a magnification 1000× (scale bar, 10 μm. Images shown are representative of three independent experiments). **(B)** The bacterial loads of intracellular *K. pneumoniae* in RAW264.7 cells. Cells (5 × 10^5^) were incubated with K7, K7R^R^, K7(Δ*GT-1*), K7(Δ*GT-2*), and K7(Δ*wcaJ*) at 37°C for 2 h. After gentamicin treatment and cell lysis, bacterial loads in each cell samples were determined by plating. Data represent the mean ± SEM of triplicate experiments. **(C)** Expression levels of pp38 and pp65 in RAW264.7 cells after incubation with *K. pneumoniae* K7, K7R^R^, K7(Δ*GT-1*), K7(Δ*GT-2*), and K7(Δ*wcaJ*). Untreated cells were used as controls. β-actin served as a loading control. **(D)** Cytotoxicity analysis of RAW264.7 cells after incubation with purified CPS from K7 (10 μg) or different *K. pneumoniae* for 2 or 12 h. Cells without any treatment were served as controls and their viability was set as 100%. The statistical differences between different treatment groups and control group were labled with # (*P* < 0.05); the statistical differences of the same treatment groups at different time periods (2 or 12 h) were labled with ^∗∗^ (*P* < 0.01), and ^∗∗∗^ (*P* < 0.001). Data represent the mean ± SEM of triplicate experiments.

Both immunofluorescence analysis and plating assay showed that almost no intracellular *K. pneumoniae* K7 in RAW264.7 cells was detected, but K7R^R^ had a high probability of being endocytosed. Like K7R^R^, a large number of K7(Δ*GT-1*), K7(Δ*GT-2*), and K7(Δ*wcaJ*) strains could be recognized and endocytosed by macrophages. However, among the three strains, K7(Δ*GT-2*) seemed to have the highest macrophage endocytosis efficiency (Figure 7A,B), indicating that K7(Δ*GT-2*) is most easily cleared by the immune cells.

NF-κB and MAPKs signaling pathways of macrophages were detected when treated with *K. pneumoniae* strains. The expression levels of pp38 in RAW264.7 cells were substantially increased after co-incubation with K7R^R^, K7(Δ*GT-1*), K7(Δ*GT-2*), or K7(Δ*wcaJ*). However, whether co-incubated with K7 or GT-deficient strains, pp65 expression levels remained unchanged in macrophages (Figure 7C). These suggest that induction of p38 phosphorylation is an important step to activate macrophages by *K. pneumoniae* after CPS loss.

The viability of the cells was measured by the amount of formazan produced by the cells (Cano et al., 2009). In spite of pure CPS from K7 (10 μg) has no obvious cytotoxic effect, capsular *K. pneumoniae* K7 has a significant killing effect on cells. After 2 h incubation with *K. pneumoniae* K7, the viability of the cells was reduced to 89.6%. Once the incubation time was increased to 12 h, the viability K7-incubated cells was only 26.2%. However, although, other non-capsulated bacteria did not show significant cytotoxicity within 2 h, the viability of the cells in these treatment groups was also decreased after 12 h. After K7(Δ*GT-1*) and K7(Δ*wcaJ*) incubation, the cell viability was 70.1 and 70.2%, respectively, which was lower than the 76.1% cell viability of K7(Δ*GT-2*)-incubated cells, suggesting that K7(Δ*GT-1*) and K7(Δ*wcaJ*) with macromolecular CPS residues could cause more severe cell damage than K7(Δ*GT-2*) (Figure 7D). However, *K. pneumoniae* K7, K7R^R^, K7(Δ*GT-1*), K7(Δ*GT-2*), and K7(Δ*wcaJ*) grew almost synchronously within 12 h in DMEM (Supplementary Figure S4), indicating that the cytotoxicity comparison of K7 and other non-capsulated *K. pneumoniae* might be able to rule out the factor of multiplication efficacy. Thus, these data further demonstrate that the cytotoxicity of *K. pneumoniae* should be attributed to other bacterial components other than CPS. However, the capsular strain relies on the capsule to resist phagocytosis of macrophages, thereby accumulating cytotoxicity by stabilizing proliferation and causing significant cell-killing effects.

## Discussion

Rough-type phage-resistant *K. pneumoniae* strains caused by the mutations of primary phage receptors (cell surface polysaccharides) are much more common than the smooth-type after co-incubation of phage and host strain (Cai et al., 2018). In the present study, with intact LPS, CPS loss was found in a rough-type phage-resistant *K. pneumoniae* strain, K7R^R^. At the same time, K7R^R^ showed virulence decline comparing with K7, which are consistent with the previous views of CPS to serve as primary phage receptor and as well as main virulence factor of *K. pneumoniae* K7 (Thurow et al., 1975; Paczosa and Mecsas, 2016). Previous studies have shown that rough-type phage-resistant mutant strains are generally accompanied by the loss of polysaccharide synthesis-associated proteins (Le et al., 2014; Li et al., 2018). However, the CPS synthesis-associated proteins that involved in K7R^R^-like anti-phage mutation are still uncertain. Therefore, differential protein profile of K7 and K7R^R^ was detected by mass spectrometry and this was further verified by immunoblot analysis. In K7R^R^, three glucosyltransferases (GT-1, GT-2, and WcaJ) encoded in the *cps* gene cluster were radically down-regulated in abundance with the reduction of CPS, suggesting that they might be associated with synthesis of cell surface polysaccharides, thereby maintaining phage sensitivity and bacterial virulence.

To further reveal the roles of GT-1, GT-2, and WcaJ in *K. pneumoniae*, the encoding genes were individually deleted. K7(Δ*GT-1*), K7(Δ*GT-2*), and K7(Δ*wcaJ*) all showed the loss of thick surface layers of CPS. Besides, all the GT-deficient strains presented no sensitivity to GH-K3. In our previous study, we identified that outer membrane protein C (OmpC) serves as secondary phage receptor for GH-K3 invasion, but its expression level was not repressed in K7R^R^ (Cai et al., 2018). Besides, deletion of *GT-1*, *GT-2*, or *wcaJ* in K7 did not cause down-regulation of OmpC (Supplementary Figure S5), suggesting that phage resistance mutation in this rough bacteria may be independent of the secondary phage receptor.

GH-K3 adsorption efficiency of periodate-treated K7 was substantially increased after prolonged incubation time (1 h) (Cai et al., 2018). However, the adsorption efficiency of K7R^R^ was almost unchanged within the same time period. Besides, unlike IO_4_^-^ treated-K7, K7R^R^ had a stable non-mucoid colony morphology and cannot restore the capsule morphology after subculture (Supplementary Figures S6A,B). Additionally, compared with K7R^R^, the expression levels of GT-1, GT-2, and WcaJ in IO_4_^-^ treated-K7 were not obviously changed (Supplementary Figure S7A), suggesting that the three glucosyltransferases may be associated with the stable inheritance of capsular traits in *K. pneumoniae* K7. However, genetic mutations have not been detected in the whole genome of K7R^R^ including *GT-1*, *GT-2*, and *wcaJ* compared to wild-type strain. Thus, in this phage-resistant strain, we hypothesized that the down-regulation of transcription and expression levels of three glucosyltransferases may result from epigenetic modifications, such as DNA methylation (Goldfarb et al., 2015).

Compared with K7, both K7R^R^ and IO_4_^-^ treated-K7 have undergone the processes of CPS loss. However, different from K7R^R^, the molecular weight of polysaccharide residues of IO_4_^-^ treated-K7 was similar to that of K7(Δ*GT-1*) and K7(Δ*wcaJ*) (Supplementary Figure S7B). Both K7(Δ*GT-1*) and K7(Δ*wcaJ*) also increased their GH-K3 adsorption efficiencies significantly by prolonging the incubation time. Besides, the phage can form blurred spots on the two strains. While, with K7R^R^-like polysaccharide residues, K7(Δ*GT-2*) also cannot improve its low GH-K3 adsorption efficiency after prolonging the incubation time. These data may indicate that GH-K3 still has certain recognition and adsorption capacity for macromolecular CPS residues caused by IO_4_^-^ treatment or *GT-1*, *wcaJ* deletion, but not for polysaccharide residues in K7R^R^ or K7(Δ*GT-2*).

In K7R^R^ and three GT-deficient strains, the sharp decline in the yield of CPS was accompanied by the production of a large amount of LPS. Additionally, the down-regulated glucosyltransferases in K7R^R^ caused moderate up-regulated of some LPS synthetases, such as WabH. In addition to the loss phage receptors, the blocking of receptors is also an important strategy of preventing phage adsorption (Labrie et al., 2010). Thus, despite GH-K3 seems to be able to adsorb to macromolecular CPS residues, its binding to the host outer membrane protein may be blocked by a new physical barrier that could be formed by the increased LPS. Previously, relationships of interdependence and transformation between CPS and LPS have been identified (Izquierdo et al., 2003; Ren et al., 2016). In the present work, the total bacterial polysaccharide contents of equal amounts (1.0 × 10^9^ CFU) of K7 and K7R^R^ were almost the same (166 vs. 168 μg), indicating the synthesis of CPS and LPS seemed to have a dynamic equilibrium relationship. Moreover, the alteration in abundance of ABC-type transport system may imply the readjusting of the energy supply in mutant cells during CPS loss and LPS production.

With extremely obvious lesions at 48 h after infection, the level of pro-inflammatory cytokines in K7-infected mice was significantly higher than in other groups of mice infected with non-capsulated bacteria. This observation at first sight, seems to be contradictory as non-capsular *K. pneumoniae* strains are expected to elicit potent inflammatory responses than the capsular strains (Yoshida et al., 2000, 2001). However, these previous results were generally based on data obtained during early stage of infection (approximately 6–24 h), whereby, the unencapsulated *K. pneumoniae* strains tend to be bound by complement C3, which greatly increases their probability of being phagocytosed by macrophages and then eliminated by cell-killing effects, such as those mediated by neutrophils or complements (Alvarez et al., 2000; Cortes et al., 2002). Similarly, *K. pneumoniae* K7R^R^, K7(Δ*GT-1*), K7(Δ*GT-2*), and K7(Δ*wcaJ*) are more susceptible to be endocytosed by RAW 264.7 cells. Due to the rapid recognition by the immune cells, capsule-deficient bacteria also can quickly induce the up-regulation of pro-inflammatory cytokines in the body. However, these bacteria are cleared by the immune responses in a short period of time, which is likely to cause the cytokine levels to rise and fall (Renois et al., 2011). Surprisingly, histopathological examination and ELISA test demonstrated that the mice in the K7(Δ*GT-1*) and K7(Δ*wcaJ*)-infected groups showed an insignificant inflammatory response after 48 h of infection. Moreover, macrophages had lower endocytosis efficiencies on K7(Δ*GT-1*) and K7(Δ*wcaJ*) than K7(Δ*GT-2*). The macromolecular CPS residues of K7(Δ*GT-1*) and K7(Δ*wcaJ*) may cause certain obstacles for macrophage phagocytosis and complement binding. Therefore, these factors may together cause the invasiveness of K7(Δ*GT-1*) and K7(Δ*wcaJ*) to be slightly lower than that of K7(Δ*GT-2*).

With the prolongation of time, the level of pro-inflammatory cytokines produced by the capsular *K. pneumoniae*-infected organism is generally in an increasing trend (Yoshida et al., 2000; 2001). In addition to impeding the complement binding, *K. pneumoniae* with K2 serotype lacks the specific mannose residue repeats are recognized by macrophages, thereby avoiding phagocytosis (Corsaro et al., 2005; March et al., 2013). We also found that *K. pneumoniae* K7 can hardly be endocytosed by RAW264.7 cells. In spite of capsular bacteria cannot induce the rapid production of pro-inflammatory cytokines in the early stage of infection, they can stably proliferate in the infected host (Lee et al., 2017). Further, the accumulated capsular strains induce the secretion of pro-inflammatory cytokines through various signaling pathways, such as TLR4 (Yang et al., 2011) or NLRP3 (Hua et al., 2015), ultimately leading to irreversible organic damage, just like the lung tissues of K7-challenged mice at 48 h after infection.

In summary, our study confirmed that the phage resistance and virulence decline of *K. pneumoniae* strains are mainly linked with the decline of CPS. By mass spectrometry and gene deletion assays, the CPS reduction is most likely associated with the downregulation of the abundance of three glycosyltransferases encoded by the *cps* gene cluster including GT-1, GT-2, and WcaJ. To further determining the specific functions of the three glycosyltransferases, wild-type genes *GT-1*, *GT-2*, and *wcaJ* were also individually reintroduced into mutant strains-K7(Δ*GT-1*), K7(Δ*GT-2*), and K7(Δ*wcaJ*) by pUC18K plasmid. However, these complement plasmids could not be stably maintained in these mutant strains. Therefore, in our follow-up studies, we will continue to seek other suitable complement plasmids to break through this technical bottleneck. Moreover, as a potential new mechanism for inducing phage resistance and virulence decline in *K. pneumoniae*, the mechanism of the decrease in the expression of three glucosyltransferases is also worthy of further exploration.

## Ethics Statement

C57BL/6J mice (Female, 18–20 g) were purchased from Liaoning Changsheng Biotechnology Co. Ltd (Benxi, Liaoning, China). All animal managements and experiments were strictly abided by the Regulations for the Administration of Affairs Concerning Experimental Animals approved by the State Council of the People’s Republic of China (1.11.1988) and approved by the Animal Welfare and Research Ethics Committee at Jilin University.

## Author Contributions

JG and WH conceived and designed the study. RC, SL, MC, ZG, YJ, HX, CZ, XW, YX, ZW, HZ, YF, CS, XF, LL, and YY performed the laboratory testing. RC, MW, and XL contributed to the LC-MS-MS analyses. RC, GW, and SR were responsible for the writing and revision of the manuscript. All authors read and approved the final manuscript.

## Conflict of Interest Statement

The authors declare that the research was conducted in the absence of any commercial or financial relationships that could be construed as a potential conflict of interest.
