# Supplementary material for: Three Capsular Polysaccharide Synthesis-Related Glucosyltransferases, GT-1, GT-2 and WcaJ, Are Associated With Virulence and Phage Sensitivity of Klebsiella pneumoniae
Source: Front Microbiol. 2019 May 28;10:1189. doi: 10.3389/fmicb.2019.01189 (PMC6546894; doi:10.3389/fmicb.2019.01189)
Supplement: Supplementary file 1 [file Data_Sheet_1.docx]

**Supplemental Materials**

**
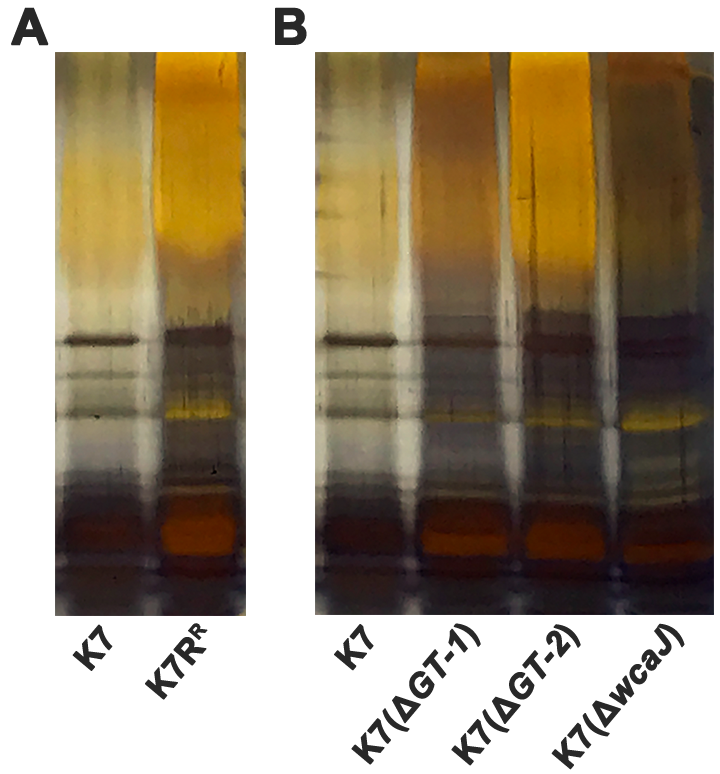
**

**FIGURE S1** LPS phenotypes of different *K. pneumoniae* strains. **(A) and (B)** LPS samples were extracted from equal amounts of *K. pneumoniae* strains (1.0×10^9^ CFU). After separated by 12% SDS-PAGE, LPS phenotypes of K7, K7R^R^, K7(Δ*GT-1*), K7(Δ*GT-2*) and K7(Δ*wcaJ*) were visualized by silver staining.


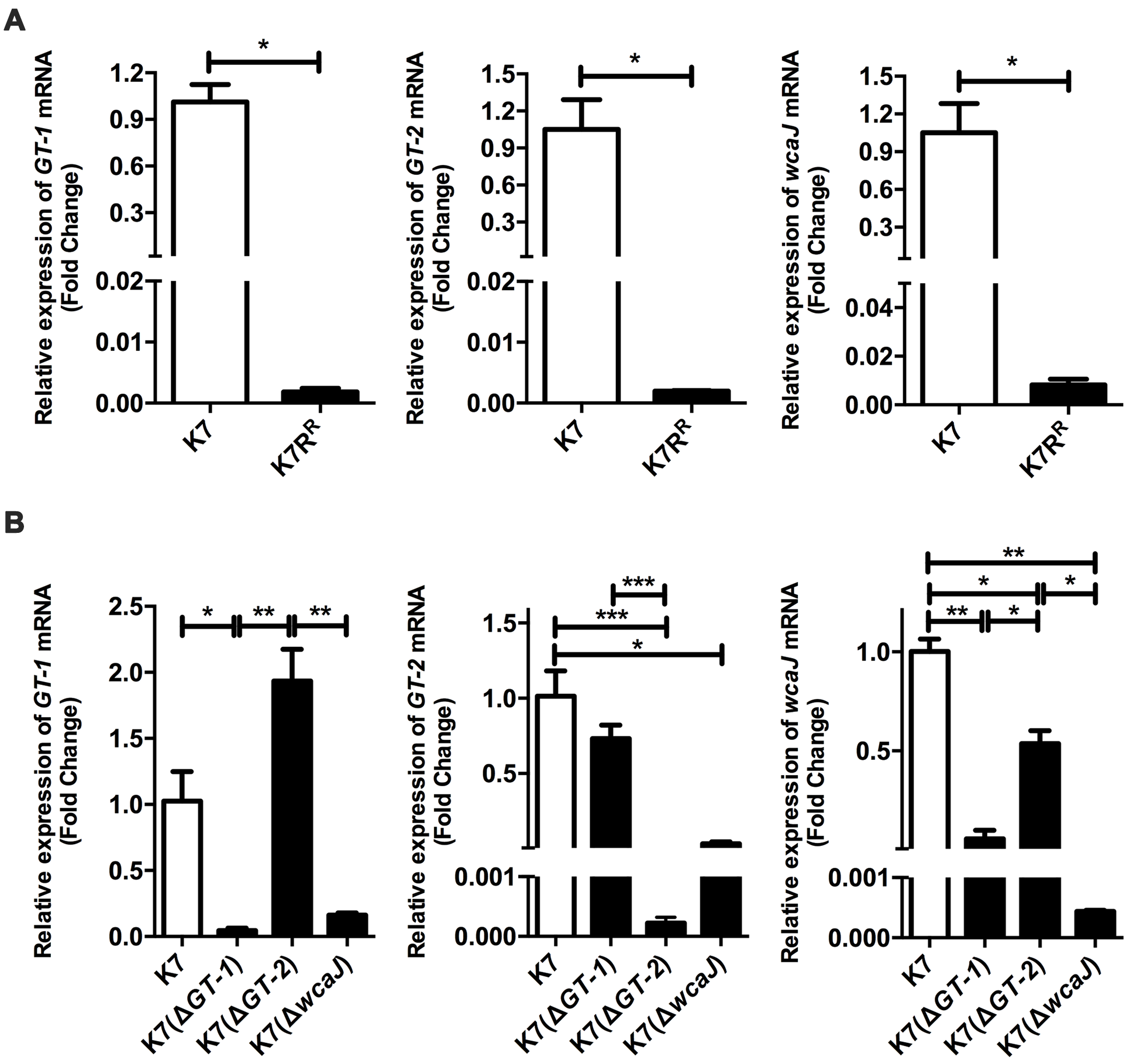


**FIGURE** **S2** The mRNA levels of *GT-1*, *GT-2* and *wcaJ.* **(A)** The mRNA transcripts levels of *GT-1*, *GT-2* and *wcaJ* in *K. pneumoniae* K7 and K7R^R^ were detected by qRT-PCR. **(B)** The mRNA transcripts levels of *GT-1*, *GT-2* and *wcaJ* in *K. pneumoniae* K7(Δ*GT-1*), K7(Δ*GT-2*) and K7(Δ*wcaJ*) were detected by qRT-PCR. *, **, and ***, significant differences at *P* < 0.05, *P* < 0.01 and *P* < 0.001, respectively. Data represent the mean ± SEM of triplicate experiments.

**
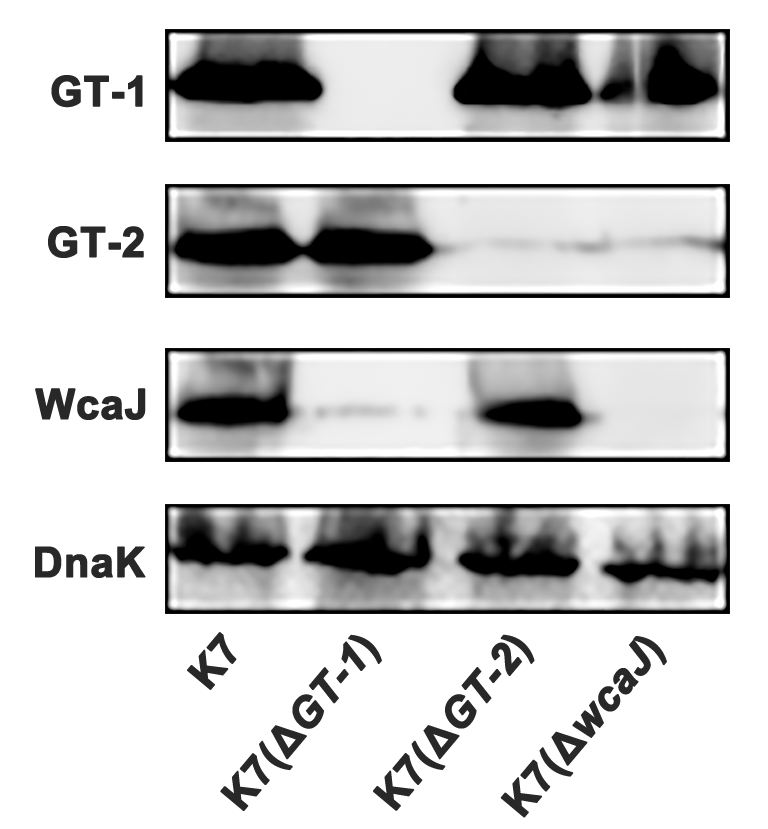
**

**FIGURE** **S3** Expression levels of GT-1, GT-2 and WcaJ. The expression levels of GT-1, GT-2 and WcaJ in *K. pneumoniae* K7(Δ*GT-1*), K7(Δ*GT-2*) and K7(Δ*wcaJ*) were detected by Western blot. K7 was used as a control. DnaK served as a loading control.

**
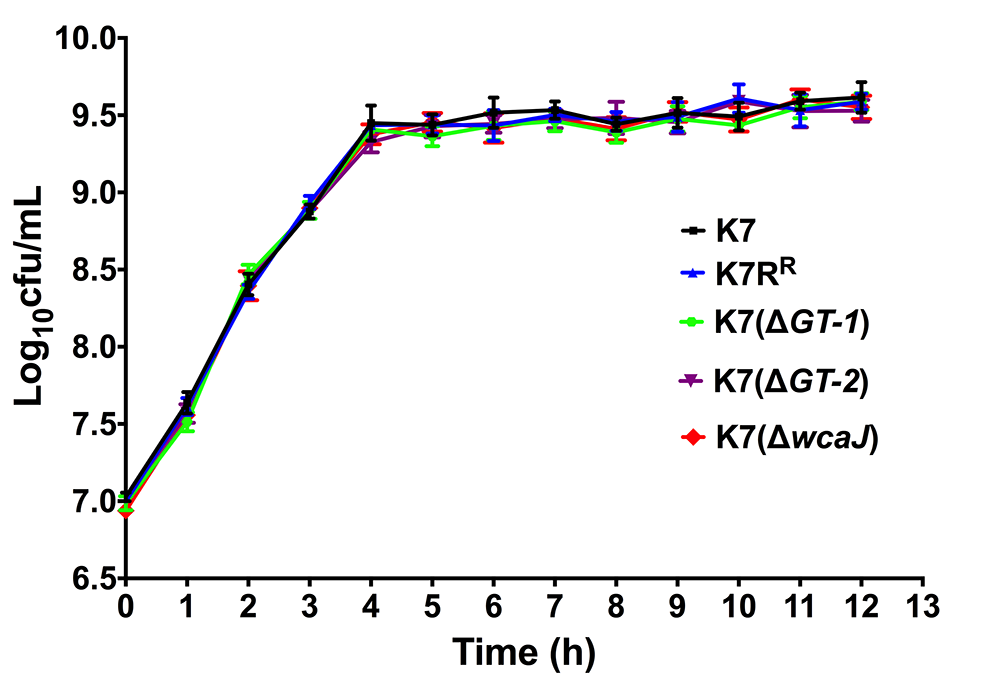
**

**FIGURE** **S4** The growth curves of *K. pneumoniae* K7, K7R^R^, K7(Δ*GT-1*), K7(Δ*GT-2*) and K7(Δ*wcaJ*) in DMEM. Different *K. pneumoniae* strains (1.0×10^6^ CFU) were inoculated in a 96-well plate containing DMEM (100 μl/well) and cultured at 37 °C for 12 h. Growth curves were constructed by plating. Statistical analysis was performed using Two-way analysis of variance (ANOVA) (No significant differences). Data represent the mean ± SEM of triplicate experiments.

**
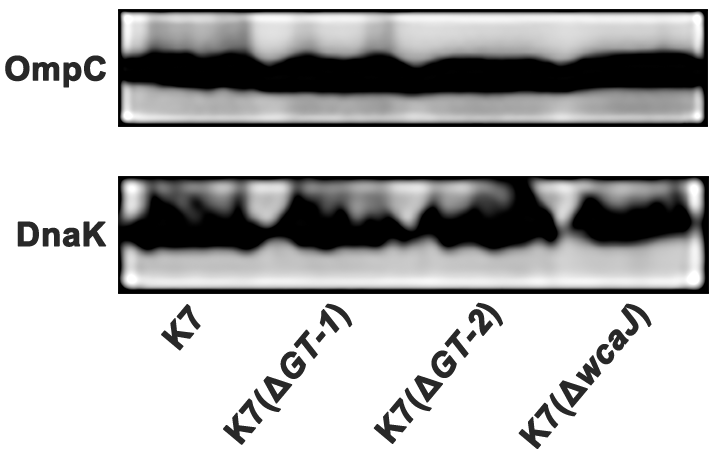
**

**FIGURE S5** Expression levels of OmpC. The expression levels of OmpC in *K. pneumoniae* K7(Δ*GT-1*), K7(Δ*GT-2*) and K7(Δ*wcaJ*) were detected by Western blot. K7 was used as a control. DnaK served as a loading control.

**
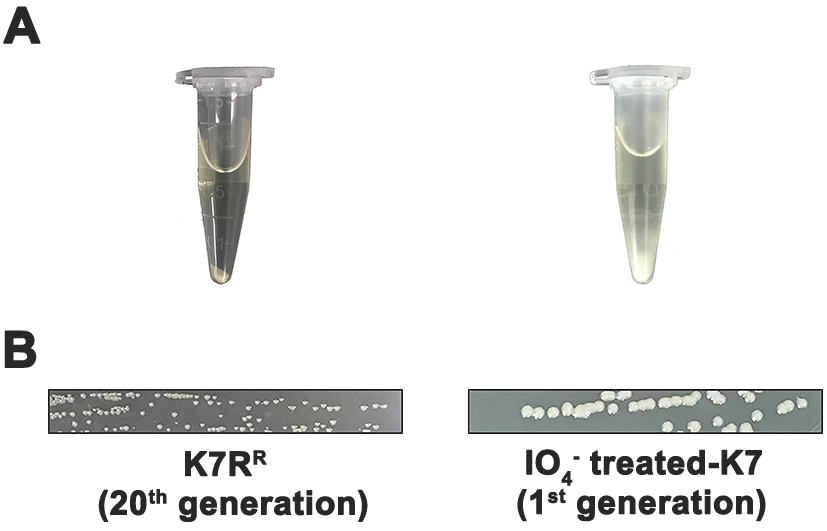
**

**FIGURE** **S6** The capsule morphology of K7R^R^ and IO_4_^-^ treated-K7 after subculture. **(A)** Centrifugation analysis of *K. pneumoniae* K7R^R^ (20^th^ generation) and IO_4_^-^ treated-K7 (1^st^ generation). The cultures were centrifuged at 10,000 ×*g* for 5 min at 4 °C. **(B)** Colony morphology of *K. pneumoniae* K7R^R^ (20^th^ generation) and IO_4_^-^ treated-K7 (1^st^ generation). Colonies of K7R^R^ and IO_4_^-^ treated-K7 were cultured on LB plates at 37 °C for 12 h after streak plating. IO_4_^-^ treated-K7 restored mucoid polysaccharide capsules after subculture, while K7R^R^ still had a stable nonmucoid morphology after 20 passages.


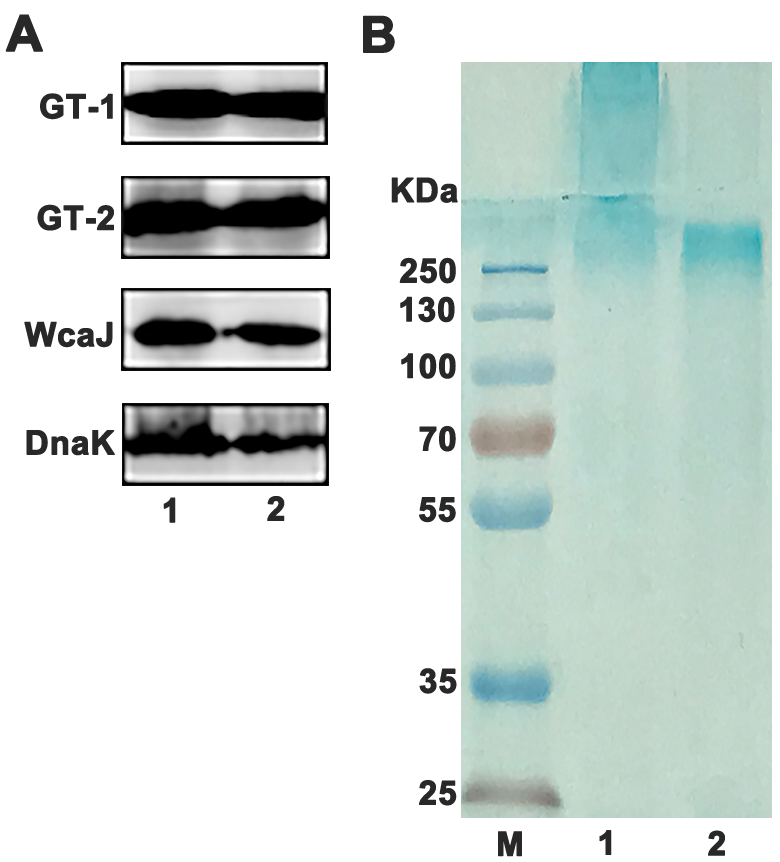


**FIGURE** **S7** GT expression levels and CPS phenotype of IO_4_^-^ treated-K7. **(A)** The expression levels of GT-1, GT-2 and WcaJ in IO_4_^-^ treated-K7 was detected by Western blot. 1: K7, which was used as a control, 2: IO_4_^-^ treated-K7. DnaK served as a loading control. **(B)** CPS samples were extracted from equal amounts of *K. pneumoniae* strains (1.0×10^9^ CFU). After separated by 12% SDS-PAGE, CPS phenotypes of K7 and IO_4_^-^ treated-K7 were visualized by alcian blue staining. 1: CPS of K7, which was used as a control, 2: CPS of IO_4_^-^ treated-K7.

**TABLE S1** Sequences of PCR primer sets.

| **For construction of plasmids with pCVD442(Km^r^) backbone** | | |
| --- | --- | --- |
| **Gene name** | **Forward primer (5’-3’)** | **Reverse primer (5’-3’)** |
| (GT-1)-5 | gtcgacacattgcaattgtgcaagtcgaatttatc | gaagatattcaagatatagatagcataccatttcctgttg |
| (*GT-1*)*-*3 | ctcaattaatgactcggccaatatctgaac | gtcgacgttgtatataatgcagtttcgctagatggac |
| (*GT-1*)*-Km* | caacaggaaatggtatgctatctatatcttgaatatcttccatatgaatatcctccttagttcctattc | gttcagatattggccgagtcattaattgaggagctgcttcgaagttccta |
| (*GT-1*)-out | ggatgaactttgttaagataccagataagtccttg | ctatgagtataaatgtgctgtagatgcaggaataag |
| (*GT-1*)-in | caacatttgagtcaagtgctaactgctt | cagaaatgtcagttaaatcgattgcggtatc |
| (*GT-2*)*-5* | gtcgacgttgtcaatatagcgataacgatcgttacag | gagaacagttagtaatagccaatgacgaagt |
| (*GT-2*)*-*3 | cgatcaaatcaacatcatgtcctgttgag | gtcgacagaaatgtcagttaaatcgattgcggtatc |
| (*GT-2*)*-Km* | acttcgtcattggctattactaactgttctccatatgaatatcctccttagttcctattc | ctcaacaggacatgatgttgatttgatcggagctgcttcgaagttccta |
| (*GT-2*)-out | gatattactattgagcatagcgacagcaaaac | gtagagttattcatacttctagagattattatctgatccatcc |
| (*GT-2*)-in | ccatcgtagatcgcttggagttctt | ccaaagggatatggctaccaagtcatatc |
| (*wcaJ*)*-5* | gtcgacatactcaatgccgttatgaaccatcttc | taagatgctgcttaagataagcattgtgc |
| (*wcaJ*)*-*3 | ttcaatcactcatttataaacaagcatatctgtcg | gtcgacgatgccgatcttctgaatgtagcatc |
| (*wcaJ*)*-Km* | gcacaatgcttatcttaagcagcatcttacatatgaatatcctccttagttcctattc | cgacagatatgcttgtttataaatgagtgattgaagagctgcttcgaagttccta |
| (*wcaJ*)-out | cttggtgaagatatctttggtaatgtcgatca | ggatatcatttcttaaacttaagaacataagagctacaatatg |
| (*wcaJ*)-in | gaggtcctacaacggacatctgac | caggtgcgttatggaatggatgga |

|  | | |
| --- | --- | --- |
| **TABLE S2** Strains and plasmids used in this work. | | |
| **Strains/plasmids** | **Genotype and properties** | **Sources/references** |
| ***E. coli*** |  |  |
| DH5α | Cloning n competent strain; F-φ80 lacZΔM15 Δ(lacZYA-arg F) U169 endA1 recA1 hsdR17(rk-,mk+) supE44λ- thi -1 gyrA96 relA1 phoA | TransGen Biotech |
| BL21(DE3) | Expression competent strain; F-ompT hsdS(rB - mB -) gal dcm(DE3) | TransGen Biotech |
| SM10 λpir | TpR SmR recA thi-1 pro hsdR-M^+^RP4: 2-Tc:Mu: Km Tn7 λpir | Laboratory stock |
| ***K. pneumoniae*** |  |  |
| K7 | Isolated from the First Hospital of Jilin University | (Gu et al., 2012) |
| K7R^R^ | Rough type GH-K3-resistant strain derived from *K. pneumoniae* K7 | (Cai et al., 2018) |
| K7(Δ*GT-1*) | An *GT-1* deletion strain of K7 | This work |
| K7(Δ*GT-2*) | An *GT-2* deletion strain of K7 | This work |
| K7(Δ*wcaJ*) | An *wcaJ* deletion strain of K7 | This work |
| **Plasmids** |  |  |
| pUC19 | Cloning vector, pUC ori, P_lac_, MCS; Amp^r^ | Laboratory stock |
| pCVD442(Km^r^) | Suicide vector Km^r^ SacB oriT oriV | Laboratory stock |
| pUC19-*GT-1* | Cloning vector with a PCR-amplified *GT-1* in the *Sal* I site; Amp^r^ | This work |
| pUC19-*GT-2* | Cloning vector with a PCR-amplified *GT-2* in the *Sal* I site; Amp^r^ | This work |
| pUC19-*wcaJ* | Cloning vector with a PCR-amplified *wcaJ* in the *Sal* I site; Amp^r^ | This work |
| pCVD442(Km^r^)-*GT-1* | Suicide vector with *GT-1* in the *Sal* I site; Km^r^ | This work |
| pCVD442(Km^r^)-*GT-2* | Suicide vector with *GT-2* in the *Sal* I site; Km^r^ | This work |
| pCVD442(Km^r^)-*wcaJ* | Suicide vector with *wcaJ* in the *Sal* I site; Km^r^ | This work |

**TABLE S3** Sequences of peptide antigens.

| **Name** | **Start-end** | **Sequence** |
| --- | --- | --- |
| GT-1 | 185-198 aa | CIKDKHIEAGFFKST |
| GT-2 | 89-102 aa | RSRLREIKLNKKYDC |
| WcaJ | 317-330 aa | CENDDKVIQATKNDI |
| DnaK | 613-626 aa | CSADAQASNAKDDDV |

aa, Amino acid. In order to strengthen the coupling with keyhole limpet hemocyanin (KLH), a "C" was added to all sequences.

**REFERENCES**

Cai, R., Wu, M., Zhang, H., Zhang, Y., Cheng, M., Guo, Z., et al. (2018). A smooth-type, phage-resistant *Klebsiella pneumoniae* mutant strain reveals OmpC is indispensable for GH-K3 infection. *Appl. Environ. Microbiol*. doi: 10.1128/aem.01585-18.

Gu, J., Liu, X., Li, Y., Han, W., Lei, L., Yang, Y., et al. (2012). A method for generation phage cocktail with great therapeutic potential. *PLoS One* 7(3)**,** e31698. doi: 10.1371/journal.pone.0031698.
